# Supplementary material for: Arbuscular Mycorrhizal Fungal Assemblages Significantly Shifted upon Bacterial Inoculation in Non-Contaminated and Petroleum-Contaminated Environments
Source: Microorganisms. 2020 Apr 21;8(4):602. doi: 10.3390/microorganisms8040602 (PMC7232219; doi:10.3390/microorganisms8040602)
Supplement: Supplementary file 1 [file microorganisms-08-00602-s001.zip › supplementary.pdf]

Supplementary material: figures S1-S4 and Details of the sequence processing pipeline

Figure S1

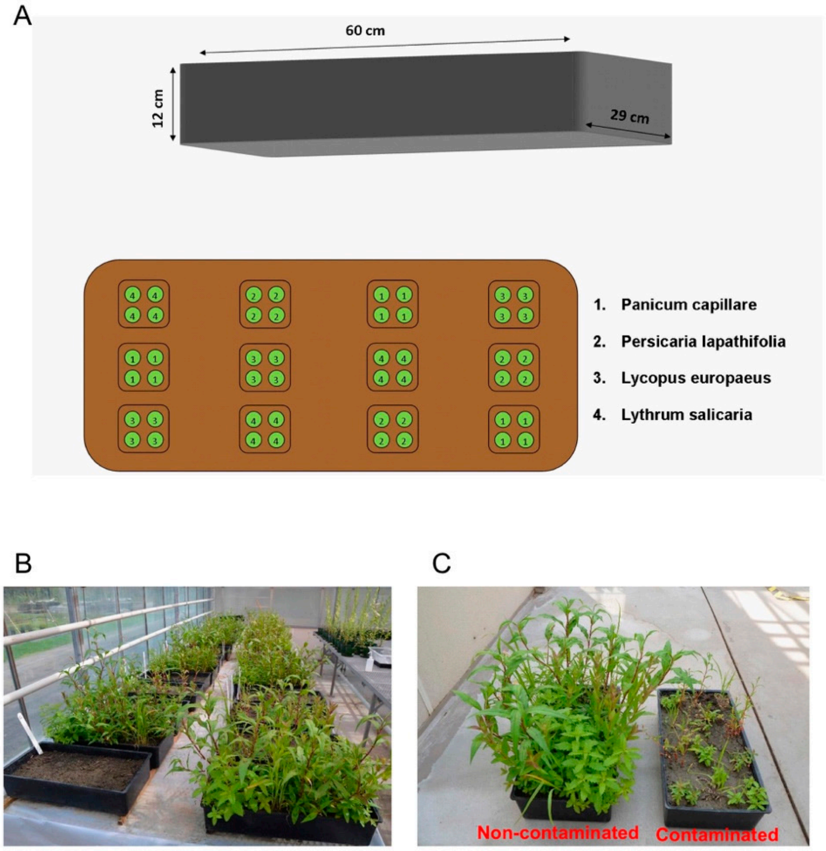

Figure S1. Planting scheme in each tray (A), experimental setup (B), and a comparison between the non-contaminated soil and contaminated sediments mesocosms (C).

Figure S2

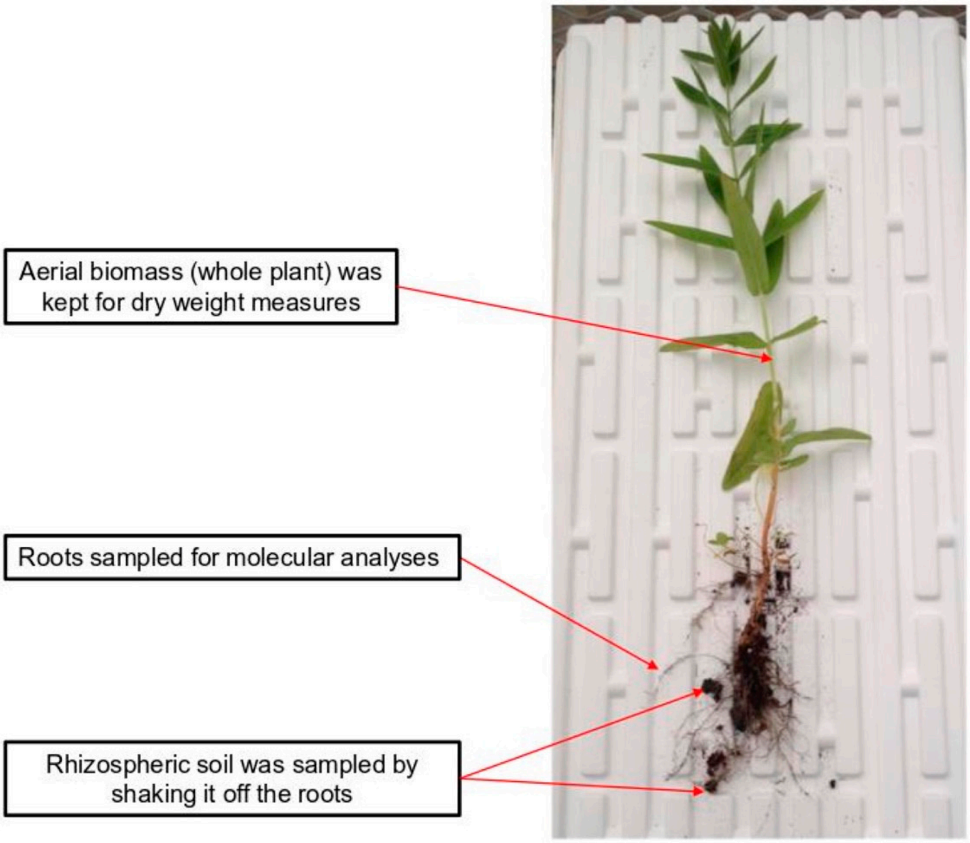

Figure S2. Plant sampling example from a *Persicaria lapathifolia*

Figure S3

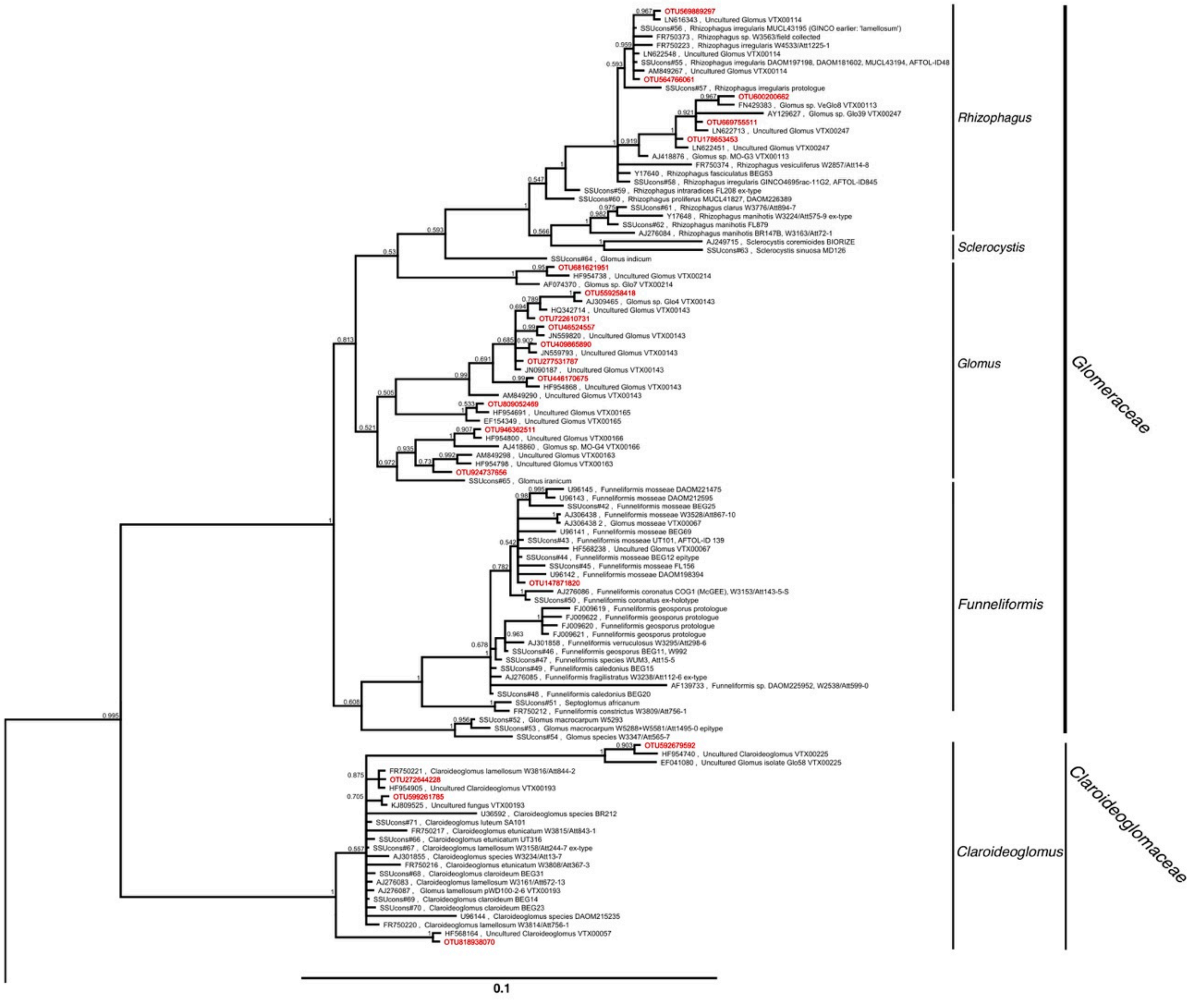
OTU569889297
LN616343 , Uncultured Glomus VTX00114
SSUcons#56 , Rhizophagus irregularis MUCL43195 (GINCO earlier: 'lamellosum')
FR750373 , Rhizophagus sp. W3563/field collected
FR750223 , Rhizophagus irregularis W4533/Att1225-1
LN622548 , Uncultured Glomus VTX00114
SSUcons#55 , Rhizophagus irregularis DAOM197198, DAOM181602, MUCL43194, AFTOL-ID48
AM849267 , Uncultured Glomus VTX00114
OTU564766061
SSUcons#57 , Rhizophagus irregularis protologue
OTU600200662
FN429383 , Glomus sp. VeGlo8 VTX00113
AY129627 , Glomus sp. Glo39 VTX00247
OTU669755511
LN622713 , Uncultured Glomus VTX00247
OTU178653453
LN622451 , Uncultured Glomus VTX00247
AJ418876 , Glomus sp. MO-G3 VTX00113
FR750374 , Rhizophagus vesiculiferus W2857/Att14-8
Y17640 , Rhizophagus fasciculatus BEG53
SSUcons#58 , Rhizophagus irregularis GINCO4695rac-11G2, AFTOL-ID845
SSUcons#59 , Rhizophagus intraradices FL208 ex-type
SSUcons#60 , Rhizophagus proliferus MUCL41827, DAOM226389
SSUcons#61 , Rhizophagus clarus W3776/Att894-7
Y17648 , Rhizophagus manihotis W3224/Att575-9 ex-type
SSUcons#62 , Rhizophagus manihotis FL879
AJ276084 , Rhizophagus manihotis BR147B, W3163/Att72-1
AJ249715 , Sclerocystis coremioides BIORIZE
SSUcons#63 , Sclerocystis sinuosa MD126
SSUcons#64 , Glomus indicum
OTU681621951
HF954738 , Uncultured Glomus VTX00214
AF074370 , Glomus sp. Glo7 VTX00214
OTU559258418
AJ309465 , Glomus sp. Glo4 VTX00143
HQ342714 , Uncultured Glomus VTX00143
OTU722610731
OTU46524557
JN559820 , Uncultured Glomus VTX00143
OTU409865890
JN559793 , Uncultured Glomus VTX00143
OTU277531787
JN090187 , Uncultured Glomus VTX00143
OTU446170675
HF954868 , Uncultured Glomus VTX00143
AM849290 , Uncultured Glomus VTX00143
OTU809052469
HF954691 , Uncultured Glomus VTX00165
EF154349 , Uncultured Glomus VTX00165
OTU946362511
HF954800 , Uncultured Glomus VTX00166
AJ418860 , Glomus sp. MO-G4 VTX00166
AM849298 , Uncultured Glomus VTX00163
HF954798 , Uncultured Glomus VTX00163
OTU924737656
SSUcons#65 , Glomus iranicum
U96145 , Funneliformis mosseae DAOM221475
U96143 , Funneliformis mosseae DAOM212595
SSUcons#42 , Funneliformis mosseae BEG25
AJ306438 , Funneliformis mosseae W3528/Att867-10
AJ306438 2 , Glomus mosseae VTX00067
U96141 , Funneliformis mosseae BEG69
SSUcons#43 , Funneliformis mosseae UT101, AFTOL-ID 139
HF568238 , Uncultured Glomus VTX00067
SSUcons#44 , Funneliformis mosseae BEG12 epitype
SSUcons#45 , Funneliformis mosseae FL156
U96142 , Funneliformis mosseae DAOM198394
OTU147871820
AJ276086 , Funneliformis coronatus COG1 (McGEE), W3153/Att143-5-S
SSUcons#50 , Funneliformis coronatus ex-holotype
FJ009619 , Funneliformis geosporus protologue
FJ009622 , Funneliformis geosporus protologue
FJ009620 , Funneliformis geosporus protologue
FJ009621 , Funneliformis geosporus protologue
AJ301858 , Funneliformis verruculosus W3295/Att298-6
SSUcons#46 , Funneliformis geosporus BEG11, W992
SSUcons#47 , Funneliformis species WUM3, Att15-5
SSUcons#49 , Funneliformis caledonius BEG15
AJ276085 , Funneliformis fragilistratus W3238/Att112-6 ex-type
AF139733 , Funneliformis sp. DAOM225952, W2538/Att599-0
SSUcons#48 , Funneliformis caledonius BEG20
SSUcons#51 , Septoglomus africanum
FR750212 , Funneliformis constrictus W3809/Att756-1
SSUcons#52 , Glomus macrocarpum W5293
SSUcons#53 , Glomus macrocarpum W5288+W5581/Att1495-0 epitype
SSUcons#54 , Glomus species W3347/Att565-7
OTU592679592
HF954740 , Uncultured Claroideoglomus VTX00225
EF041080 , Uncultured Glomus isolate Glo58 VTX00225
FR750221 , Claroideoglomus lamellosum W3816/Att844-2
OTU272644228
HF954905 , Uncultured Claroideoglomus VTX00193
OTU599261785
KJ809525 , Uncultured fungus VTX00193
U36592 , Claroideoglomus species BR212
SSUcons#71 , Claroideoglomus luteum SA101
FR750217 , Claroideoglomus etunicatum W3815/Att843-1
SSUcons#66 , Claroideoglomus etunicatum UT316
SSUcons#67 , Claroideoglomus lamellosum W3158/Att244-7 ex-type
AJ301855 , Claroideoglomus species W3234/Att13-7
FR750216 , Claroideoglomus etunicatum W3808/Att367-3
SSUcons#68 , Claroideoglomus claroideum BEG31
AJ276083 , Claroideoglomus lamellosum W3161/Att672-13
AJ276087 , Glomus lamellosum pWD100-2-6 VTX00193
SSUcons#69 , Claroideoglomus claroideum BEG14
SSUcons#70 , Claroideoglomus claroideum BEG23
U96144 , Claroideoglomus species DAOM215235
FR750220 , Claroideoglomus lamellosum W3814/Att756-1
HF568164 , Uncultured Claroideoglomus VTX00057
OTU818938070
Rhizophagus
Sclerocystis
Glomus
Funneliformis
Claroideoglomus
Glomeraceae
Claroideoglomaceae
0.1

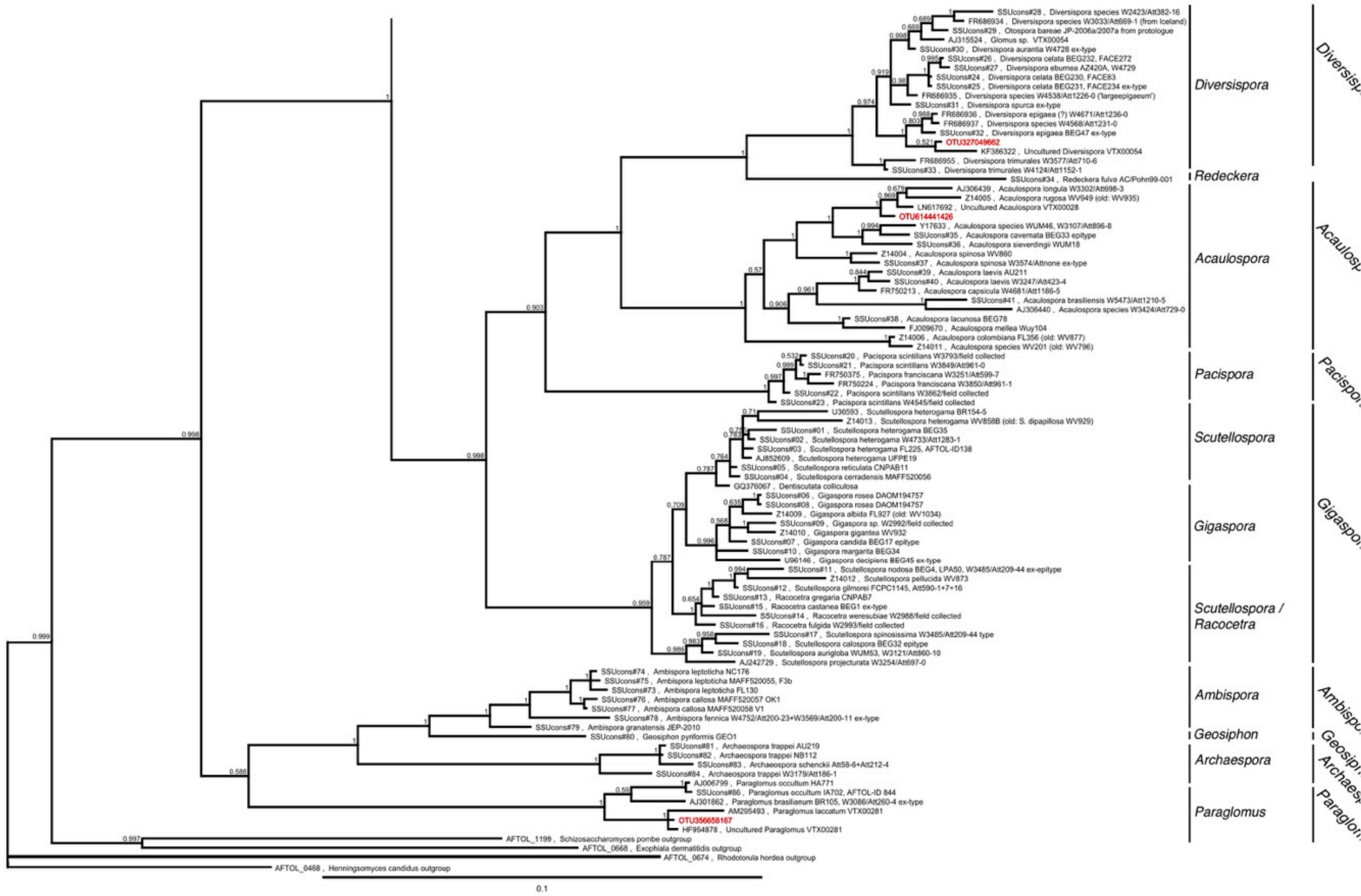

Figure S3. Bayesian phylogenetic tree based on nuclear small subunit (SSU) rDNA consensus sequences showing the distribution of the 23 OTUs recorded in this experiment (red labels) among the *Glomeromycota* tree. Sequence data were analyzed with the SSU sequences (black labels) from Krüger et al. (2012) and the closest matches recovered from MaarjAM database. The numbers on the nodes represent the Bayesian posterior probabilities. The scale represents the branch length corresponding to expected substitutions per site.

Figure S4

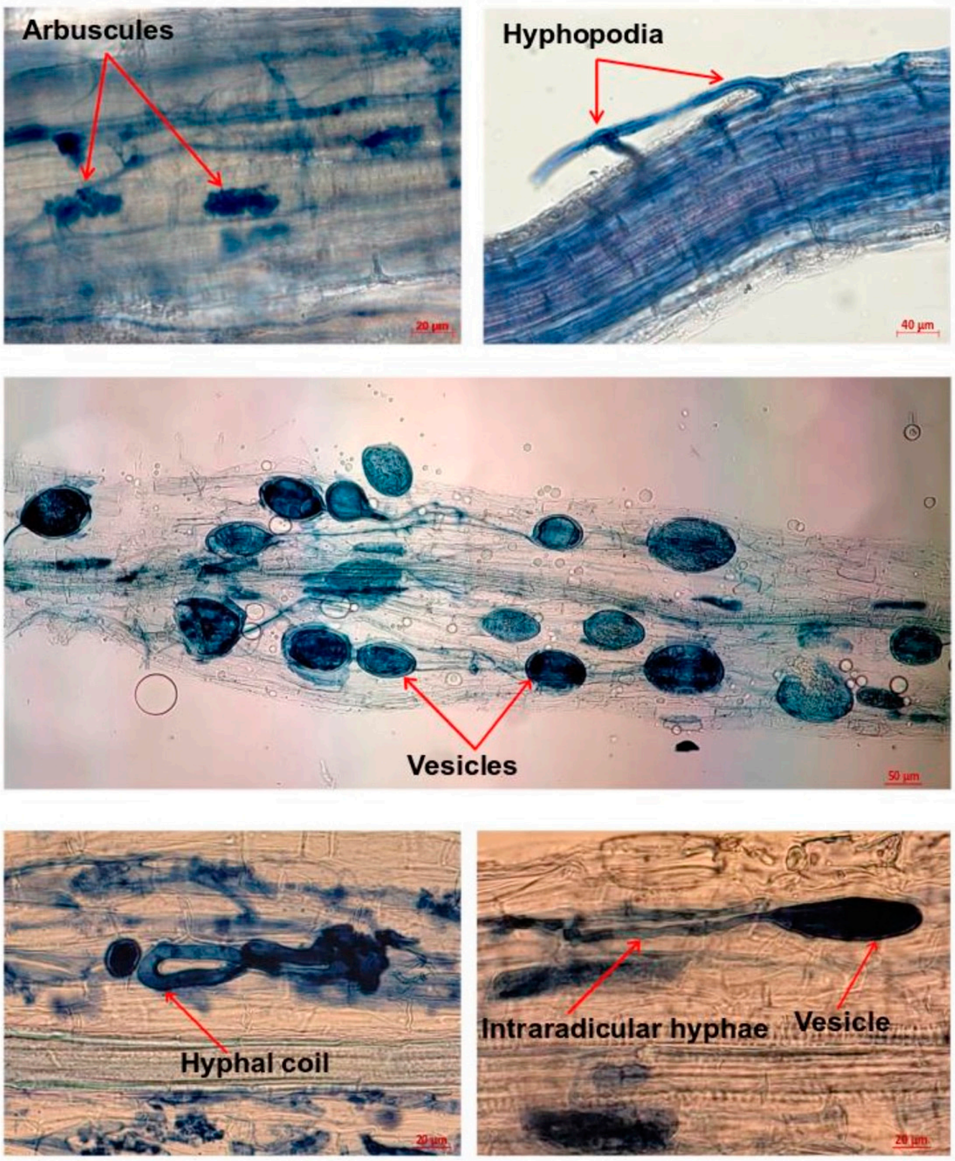

Figure S4. Mycorrhizal structures observed in the plant roots under light microscopy
